# When Chinese Employees Speak Up: The Experience of Organizational Trust and Authenticity Enhances Employees’ Voice Behavior

**DOI:** 10.3390/ijerph192315726

**Published:** 2022-11-25

**Authors:** Xingyun Liu, Lili Song, Jiewen Zheng, Yong Wang

**Affiliations:** 1 Key Laboratory of Adolescent Cyberpsychology and Behavior, Ministry of Education, Key Laboratory of Human Development and Mental Health of Hubei Province, School of Psychology, Central China Normal University, Wuhan 430079, China; 2 CAS Key Laboratory of Mental Health, Institute of Psychology, Beijing 100101, China; 3 Department of Psychology, University of Chinese Academy of Sciences, Beijing 100049, China; 4 The Research Center for Psychological Education, University of International Relations, Beijing 100091, China

**Keywords:** organizational trust, employee authenticity, voice behavior

## Abstract

Voice behavior is important for innovation, mistake prevention and organizational performance. Because organizational trust increases employees’ possibility of disclosing their real inner ideas, we examined the relationships between organizational trust and voice behavior, focusing especially on the avenue of impelling people to feel a higher level of authenticity. We used multiple methods to analyze the relationship. First, we used two separate surveys (Studies 1a and 1b) with different questionnaires and populations to analyze the mediation relationship and generalize the results. Then, to test the causal path, an experiment (Study 2a) in which organizational trust was manipulated was designed. The results showed that employees’ authenticity mediated the relation between organizational trust and voice behavior. To further test the causal effect of authenticity in the above mediation, authenticity was manipulated in another experiment (Study 2b). The results illustrated that higher levels of authenticity directly led to higher levels of voice behavior. These results support the hypothesis and expound on the psychological mechanism of how organizational trust increases voice behavior. The theoretical and practical implications of these findings are discussed.

## 1. Introduction

Over the past 30 years, increasing attention has been given to voice behavior [1,2,3,4]. Existing research has confirmed that voice behavior has a great influence on innovation, mistake prevention and organizational performance [5,6,7,8]. Why do some organizations allow employees to speak up more freely than others? Furthermore, why do some employees have a higher level of voice behaviors than others, even within the same organization? Existing research foregrounds numerous environmental factors, such as group voice climate [9] and workplace fun [10]. Individual differences have also been examined, such as self-esteem [1] and personality traits [11,12].

With the vigorous development of positive psychology, researchers are paying increasing attention to individuals’ character strengths and virtues, such as authenticity, creativity, kindness, and fairness [13]. Authenticity has been connected to optimal human functioning and has been shown to promote both intrapersonal and interpersonal wellbeing [14,15,16,17,18]. Researchers have also proven the appropriateness of authenticity at work both for leaders and employees. For example, employee authenticity is associated with positive work attitudes and behaviors, such as work engagement, job satisfaction, performance, and voice behavior across many cultures, which further promotes organizational effectiveness [19,20,21,22].

Despite widespread agreement on the benefits of employee authenticity, relatively few studies have investigated how it can be fostered. Theories and empirical research both supported the fact that organizational trust could improve the level of consistency between inner states and behavior [23,24]; therefore, enhancing organizational trust may be an effective way to improve the congruence between employees’ inner desires and their behaviors, i.e., they feel a higher level of authenticity and therefore display more voice behaviors.

In addition to the aforementioned research gap, promoting authenticity may not always be a priority for certain cultures, as they are more prone than others to embrace inauthenticity. People from East Asian cultures, for example, place a high value on restraint as a means of maintaining interpersonal harmony, just as the Chinese do. Even though existing research suggests that Chinese employees could benefit from being authentic [19], more research is needed to investigate the antecedents and consequences of authenticity among Chinese employees. Moreover, because existing studies are mainly cross-sectional and use data from the same sources, they cannot rule out common method bias [25] or establish the causal chain between those variables. Therefore, we combined questionnaires and experimental manipulation in the current study to fill in the above research gaps.

### 1.1. Organizational Trust and Voice Behavior

Organizational trust can be defined as the willingness of organizational members to be vulnerable to the actions of the organization and of other members based on the expectation that the organization and other members will perform a particular action that is important to themselves, regardless of the ability to monitor or control the organization or other members [23,26]. McCauley and Kuhnert [27] introduced a tripartite model of organizational trust, targeted toward employees. They proposed that organizational trust consisted of three parts: trust in colleagues, trust in leaders and trust in the whole organization. Organizational trust can also be classified into cognitive-based trust and affective-based trust based on the different psychological processes. Cognition-based trust originates from the assessment of others’ track record, for example, the competency and dependability that colleagues or leaders have shown in the past. Affect-based trust, on the other hand, emerges because of social interactions and indicates confidence in others as well as care for others’ benefits [28].

Voice behavior refers to “speaking out and challenging the status quo, with the intent of improving the situation” in the current research [1]. Hirschman [29] first proposed the concept as one of three possible reactions to dissatisfaction in the workplace. A consensus has been reached that many factors influence whether an individual displays voice behavior. These factors can be classified into different levels: individual-level factors, such as personal control [30] and cognitive-style preferences [31]; contextual-level factors, such as organizational trust [32], leadership behavior [33] and organizational hierarchy [34]; and the interaction between individual-level and contextual-level factors, for example, the interaction between self-monitoring, top management openness and trust in the supervisor [35].

Voice behavior may be classified into different categories. For example, Liang et al. [36] classified voice behavior into the promotive voice and prohibitive voice. The promotive voice is future-oriented and focuses on creatively improving the overall functioning of organizations. In contrast, the prohibitive voice is present-oriented and concentrates on the shortcomings of work practices, incidents, or employee behavior. Based on the Chinese traditional Confucian doctrine of the mean (Zhong Yong) that encourages obtaining a holistic view of the situation before responding rather than acting on instincts, Duan and Ling [37] proposed that voice behavior can be dichotomized as the overall-oriented voice and self-centered voice. The overall-oriented voice reflects individuals’ need to integrate and connect with the circumstances, focusing on union with others (i.e., O motivation). Employees who adopt this type of voice can fully consider the interests of all parties, tactfully express their suggestions, provide sufficient reasons for voice, take their superiors’ feelings into account and make concessions when necessary. In contrast, the self-centered voice reflects the individuals’ urge for self-enhancement (i.e., S motivation). Employees adopting this type of voice mainly consider problems from their own perspective, directly put forward their suggestions without considering others’ feelings too much, and they will not easily make compromises. Furthermore, the empirical evidence from Chinese employee participants shows that the Chinese traditional thinking style Zhongyong was positively related to overall-oriented voice but negatively related to self-centered voice [37].

In recent decades, scholars have paid increasing attention to the relationship between organizational trust and voice behavior, and they have all reached a consensus that organizational trust could significantly predict voice behavior [32,34,35,38]. However, relatively little research explores the psychological mechanism behind as well as the current situation in the sample of Chinese employees. Such exploration is important since it could provide a deeper understanding of how organizational trust promotes voice behaviors among Chinese employees.

### 1.2. The Mediating Role of Employee Authenticity

Authenticity refers to “the unobstructed operation of one’s true, or core, self in one’s daily enterprise” [39]. According to the person-centered conception of authenticity theory, individual authenticity was conceptualized from three dimensions: authentic living, self-alienation and accepting external influence [18]. Specifically, authentic living indicates that one can live in line with their core beliefs and values, i.e., being true to themselves. Self-alienation is a subjective feeling of not knowing oneself or touching the true self. Accepting external influence refers to the degree to which one accepts others’ influence or has to act according to others’ expectations. In this tripartite model of authenticity, authentic living is an indicator of high levels of individual authenticity, while self-alienation and accepting external influence are indicators of low levels of individual authenticity [18].

One’s authenticity can be affected by the external environment. Following this line, Van den Bosch and Taris [21] introduced this model into the workplace, targeted employees, and developed a state-like measure of authenticity in the work context. When the environment is trustworthy, the proportion between inner deposition and external performance would be improved, i.e., people will be prone to experience a higher level of authenticity than in an environment that is untrustworthy. Moreover, previous research has demonstrated that organizational trust can enhance employees’ perceptions of psychological safety [40]. When employees perceive more psychological safety, they will be more willing to show their true attitudes, dispositions, and feelings; that is, they will be more authentic at work. In addition, according to the State Authenticity as Fit to the Environment (SAFE) model [16], when environmental cues are aligned with individuals’ values, they will feel goal fit and thus promote motivational fluency; when they are well-recognized by others, they will feel social fit and thus enhance interpersonal fluency. Prior research demonstrates that trust is strongly associated with goal congruence [41] and that trust itself is a positive indicator of interpersonal communication [23]. Therefore, organizational trust can be regarded as a crucial environmental indicator that will further make employees feel at ease to be authentic at work.

Meanwhile, employee authenticity can yield more voice behaviors. Generally, employees with high levels of authenticity apply problem-oriented coping styles rather than maladaptive coping styles (e.g., denial, neglect or exit). Therefore, to actively solve the problem or modify the source of the threat, they are more likely to express their true opinions or beliefs according to their judgements and exhibit more voice behaviors in their roles [14]. Indeed, research has demonstrated that regardless of whether responding to hypothetical or actual problematic workplace events, employees who felt more authentic reported more voice behaviors and less silence. Furthermore, this phenomenon still existed after controlling for a bulk of demographic factors and organization-based forces [42]. Another study by Song et al. [19] also revealed that employee authenticity was positively related to both prohibitive and promotive voice. Hence, when employees work in a trustworthy organization, they tend to experience authenticity and thus feel free and safe to speak up for the purpose of overcoming shortcomings and improving organizational functioning. Following this line, we propose that employee authenticity mediates the relationship between organizational trust and their voice behavior, i.e., organizational trust enhances voice behavior by increasing employee authenticity.

### 1.3. Overview

Existing research has pointed out that organizational trust can impel employees to reveal their inner ideas. Additionally, a higher level of authenticity is related to more voice behaviors. Therefore, we hypothesized that authenticity mediates the relationship between organizational trust and voice behavior and examined this relationship in Chinese employees. To verify this hypothesis, we conducted four consecutive studies. Study 1a used multiple surveys to measure organizational trust, employee authenticity, and voice behavior. This study is aimed at directly testing mediation. Different dimensions may have an impact on the relationship between variables since they represent variations in the constructions of variables (such as organizational trust and voice behavior mentioned above). The link between variables can be illustrated in more detail by measuring variables with different dimensions. Therefore, Study 1b used divergent questionnaires with distinct dimensions of the same variables in a different population to replicate the results. Apart from the survey, we also want to verify the mediation through experiments. We manipulated the experience of organizational trust in Study 2a. Then, we analyzed its influence on state authenticity and voice behavior and whether the mediation still existed in this circumstance. Next, Study 2b was designed to complete the causal effect examination by testing whether manipulated authenticity could directly change the level of voice behavior. Therefore, because we both measured and manipulated the mediator, i.e., the level of authenticity, we tested the hypothesized causal chain (organizational trust → authenticity → voice behavior) with the integration of Studies 2a and 2b [43]. To the best of our knowledge, this is the first study to provide evidence for the causal effect between organizational trust, authenticity, and voice behavior. The project received ethical approval from the Institutional Review Board of the Institute of Psychology, Chinese Academy of Sciences, with the ethics approval number H19031. Participants were informed that their data would be kept anonymous and confidential. Their information would be utilized solely for academic research, and no colleagues or personnel unrelated to academic research would have access to individual data.

## 2. Study 1a: Organizational Trust, Authenticity, and Voice Behavior

In Study 1, we first verified the hypothesis that authenticity mediates the relationship between organizational trust and voice behavior through the use of questionnaire surveys.

### 2.1. Method

Participants and procedure. We recruited 214 employees from an online website dedicated to participant recruitment. Participants received RMB 7 (nearly 1 dollar) for filling out the questionnaires. A total of 18 participants were excluded because they did not take the questionnaires seriously, such as identical answers for all items or too much missing data, which resulted in a final sample of 196 employees (106 women and 90 men). The participants were relatively young (50% of them were between the ages of 30 and 39), and 67.35% had more than five years of work experience. Additionally, they represented different types of enterprises, with 22.96% being from state-owned enterprises, 15.31% from foreign enterprises, 28.57% from private enterprises, 25.52% from self-employed enterprises and 7.65% from joint enterprises. The participants varied substantially by demographic characteristics.

Measures. Unless otherwise stated, participants were measured with a 5-point Likert scale (1 = strongly disagree, 5 = strongly agree). Because previous studies documented that gender, age, length of service, educational level, type of enterprise, position, income, marital status, and weekly working hours may correlate with voice behavior [1,42] or organizational trust [26,27], we used these factors as control variables in this study.

Organizational trust. Organizational trust was assessed via the trust in organization scale [44]. This 13-item scale has three dimensions: trust in the organization (5 items), trust in leaders (4 items) and trust in colleagues (4 items). A sample item is “I believe the company can give employees benefits and take care of employees”.

Employee Authenticity. Employee authenticity was assessed via the Individual Authenticity Measure at Work (IAM Work) for Chinese Employees [45]. This scale consists of 21 items and 3 dimensions: authentic living (10 items), self-alienation (6 items), and accepting external influence (5 items) with 7-point scales (1 = strongly disagree, 7 = strongly agree). ‘I am true to myself at work in most situations’ is a sample item.

Voice behavior. Voice behavior was measured with the employee voice behavior scale [37]. This scale includes 11 items and 2 dimensions: overall-oriented voice (6 items) and self-centered voice (5 items). ‘Always choose the right place and time to give advice to colleagues or leaders’ is a sample item.

### 2.2. Results and Discussion

Table 1 presents the means, standard deviations, and correlations of all variables.

We conducted a series of regressions to test our hypothesis. As shown in Table 2 (Model 2), voice behavior was significantly predicted by organizational trust after controlling for gender, age, length of service, educational level, enterprise type, position, monthly income, marital status and weekly working hours (*B* = 0.43, *SE* = 0.06, *t* = 7.66, *p* < 0.01). Furthermore, our regression results show that organizational trust significantly predicted employee authenticity after including the same control variables (*B* = 0.74, *SE* = 0.12, *t* = 6.40, *p* < 0.01). When employee authenticity was introduced into Model 3, it was found that employee authenticity significantly predicted voice behavior (*B* = 0.09, *SE* = 0.04, *t* = 2.52, *p* < 0.05), indicating a mediation effect. Finally, we tested the indirect effect of employee authenticity with the assistance of bootstrap procedures with 1000 iterations. The results show that there was a significant indirect effect of authenticity on the relationship between organizational trust and voice (indirect effect = 0.07, 95% CI = [0.002, 0.13], which supports our hypothesis.

## 3. Study 1b: Retests of the Chain with a Different Population and Questionnaires

With a different population and surveys, we double-checked our hypothesis in Study 1b. Study 1a showed that enterprise type and position have a significant effect on voice behavior; thus, in Study 1b, we recruited participants from the same company and the same title. Moreover, we first measured organizational trust, and two months later, we measured authenticity and voice behavior.

### 3.1. Method

Participants and procedures. A total of 215 employees from a biological manufacturing company in Southeast China finished the first wave, and 182 of them finished the second wave (129 women and 53 men). The average age was 33.99 years (*SD* = 4.61). On average, they had worked 11.43 years (*SD* = 4.78) for the company and 7.07 years (*SD* = 4.87) in the current position. Most of the participants were married (92.31%), had graduated from high school or junior college (94.51%) and earned between RMB 1500 and RMB 5000 (nearly USD 209 and USD 698 per month (94.51%).

Measures. Unless otherwise noted, participants were measured with a 7-point Likert scale (1 = strongly disagree, 7 = strongly agree).

Organizational trust. Organizational trust was assessed via the Cognition- and Affect-based Trust Scale [28]. This 8-item scale has 2 dimensions: cognitive trust (4 items) and affective trust (4 items). ‘You can rely on them to do a major portion of the group work’ is a sample item.

Employee Authenticity. Employee authenticity was assessed using the same scale as in Study 1.

Voice behavior. Voice behavior was measured with the scale by Liang et al. [36]. This scale includes 2 dimensions: promotive voice (5 items) and prohibitive voice (5 items). ‘I proactively develop and make suggestions for issues that may influence my team’ is an example item. Each item was rated on a scale of 1 (almost never) to 5 (almost always).

### 3.2. Results and Discussion

Table 3 presents the means, standard deviations, and correlations of all variables.

Again, we conducted a series of regressions to test our hypothesis. As shown in Table 4 (Model 2), organizational trust significantly predicted voice behavior after controlling for gender, age, tenure in the current company, tenure in the current position, education, monthly income and marital status (*B* = 0.30, *SE* = 0.04, *t* = 4.09, *p* < 0.001). Moreover, the regression results show that organizational trust significantly predicted employee authenticity after including the same control variables (*B* = 0.19, *SE* = 0.05, *t* = 2.52, and *p* = 0.013). After introducing employee authenticity into Model 3, it was found that employee authenticity significantly predicted voice behavior (*B* = 0.30, *SE* = 0.05, *t* = 5.56, and *p* < 0.001), indicating a mediation effect. Finally, the indirect effect of employee authenticity was tested with the assistance of bootstrap procedures with 1000 iterations. The results show that the indirect effect of authenticity in the relationship between organizational trust and voice was significant (indirect effect = 0.03, 95% CI = [0.002, 0.07], which further supports our hypothesis and indicates that our results can be retested by different populations and questionnaires.

## 4. Studies 2a and 2b: Experimental Tests of the Mediation

We used Studies 2a and 2b to further test the mediation effect of authenticity between organizational trust and voice behavior. To achieve this, we first conducted a survey in which participants needed to respond to hypothetical scenarios in Study 2a. In this study, organizational trust was manipulated, and the mediation relation was tested. However, due to the measurement nature of authenticity and voice behavior, we could not directly verify the causal effect between organizational trust, authenticity and voice behavior. Therefore, we experimentally manipulated authenticity in Study 2b to improve and perfect this analysis [43].

## 5. Study 2a: Manipulated Organizational Trust

In this study, we hypothesized that employees who received high-organizational trust manipulation would have higher levels of authenticity and more voice behavior than those who received low-organizational trust manipulation. Moreover, we also verified the mediation effect of authenticity between organizational trust and voice behavior.

### 5.1. Method

One hundred twenty-seven employees (69 women, 58 men; *M_ag_*_e_ = 31.43, *SD_ag_*_e_ = 6.41) were recruited from the same online website as in Study 1a. Participants could obtain RMB 8.5 (nearly USD 1.20) for their work. To prevent the participants from figuring out the objective of the study, they were told that they would join in several distinctive experiments with some other scales. In the first experiment, we adopted the scenario technique to manipulate organizational trust by randomly assigning participants to a scenario describing one organization that can be trusted and the other that cannot. It should be noted that in this instance, trust manipulation referred to asking participants to imagine how they would respond to one of the two randomly assigned scenarios as opposed to encouraging them to have confidence or not in the organization they were working for. The exact wording of the scenarios was as follows:

*“Work is an important part of life, and you are now a staff member of a particular company.*

*You believe that the company has [no] the ability to give employees benefits and to take care of employees. Even if the future situation is not certain, the company will not [will] do harm to employees. As far as you know, most of your colleagues think that this company is [not] trustworthy and are [not] confident about the development of your company. Finally, you believe that the company can [not] adhere to and implement its management policies.*

*Regarding company leaders, you believe that they [do not] can perform their duties. They are [not] usually sincere about the staff’s opinion. They are [not] honest with you. They can [not] treat you fairly. More importantly, no matter what happens, you believe that your leader will [not] give you support and assistance.*

*Regarding colleagues, you believe that they are [not] competent in their work. You believe that most of your colleagues can [not] fit their deeds to their words at work. In addition, when you have difficulties at work, you believe that you can [not] get help from your colleagues. When you are too busy, you also believe that your colleagues will [not] help you.”*

The participants were asked to envision their job experience in such a company and write down their sentiments in 50 words or more, the greater the better. After finishing writing, they were asked to estimate to what degree they could trust the imaginary company (from 1 = not at all to 7 = very much). Next, the participants completed the same authenticity scale as in Study 1. The alpha for the full scale was 0.91. Finally, we used the well-validated scenario technique by Knoll and Van Dick [42] to assess employee voice behavior. There were two scenarios of different intensities that could increase the research’s external validity. Scenario 2 was more serious than Scenario 1, which could cause more damage to organizations, even customers. More information can be found in the article of Knoll and Van Dick [42]. Voice behavior was assessed with the same three items as in Knoll and Van Dick’s [42] study on a 5-point scale (1 = strongly disagree, 5 = strongly agree). An example item is as follows: “I would address the problem even if speaking up entailed disadvantages”.

### 5.2. Results and Discussion

To check the manipulation effect, we first compared the two groups’ degrees of self-reported trust. As expected, the participants in the high-trust condition (*M* = 6.31, *SD* = 0.68) expressed having a higher level of organizational trust than those in the low-trust condition (*M* = 2.24, *SD* = 1.66, *t* = 17.93, *p* < 0.001, *d* = 3.21). Then, two independent judges who were blinded to the experimental design were asked to estimate how much organizational trust the participants expressed in their essays on a 7-point scale (1 = low, 7 = high; *r* = 0.87). Again, the participants in the high-trust group (*M* = 5.53, *SD* = 0.74) expressed a higher level of organizational trust than those in the low-trust group (*M* = 2.37, *SD* = 0.80, *t* = 23.15, *p* < 0.00, *d* = 4.10).

Consistent with our hypothesis, the high-organizational trust group (*M* = 4.75, *SD* = 0.77) reported more state authenticity than the low-organizational trust group (*M* = 4.47, *SD* = 0.86, *t* = 1.92, *p* < 0.05, *d* = 0.34). The situation was the same when dealing with voice behavior: the high-organizational trust group (*M* = 4.10, *SD* = 0.53) reported having more state voice behavior than the low-organizational trust group (*M* = 3.37, *SD* = 0.83, *t* = 4.25, *p* < 0.001, *d* = 1.05). Moreover, authenticity significantly predicted voice behavior (*B* = 0.25, *SE* = 0.08, *p* < 0.001), and the 95% BCa CI (0.00, 0.17) illustrated that state authenticity was a mediator between organizational trust and voice behavior. The results were consistent when the two scenarios with different intensities were analyzed separately. Specifically, for Scenario 1, the high-organizational trust group (*M* = 4.02, *SD* = 0.65) reported having more state voice behavior than the low-organizational trust group (*M* = 3.42, *SD* = 0.95, *t* = 4.12, *p* < 0.001, *d* = 0.74). For Scenario 2, the high-organizational trust group (*M* = 4.18, *SD* = 0.62) reported having more state voice behavior than the low-organizational trust group (*M* = 3.73, *SD* = 0.86, *t* = 3.44, *p* = 0.001, *d* = 0.61).

## 6. Study 2b: Manipulated Authenticity

This study was designed to finish the second part of the proposed causal chain (authenticity → voice behavior). In this study, we manipulated authenticity to determine whether it has a causal effect on voice behavior.

### 6.1. Method

One hundred twenty-two employees (63 women, 59 men; *M_ag_*_e_ = 32. 34, *SD_ag_*_e_ = 6.45) were recruited from the same website as mentioned above. They obtained RMB 7.5 (nearly USD 1.06) for their work. Participants were randomly assigned to an authentic situation or an inauthentic one. They were asked to recollect and write about in such a situation how they were feeling. The exact words used to wake up their memory were as follows:

*“Please recall a particular incident in which you felt authentic [inauthentic]. By authentic [inauthentic], we mean a situation in which you were [not] true to yourself and experienced yourself as [not] behaving in accordance with your true thoughts, beliefs, personality, or values. Try to relive this situation in your imagination. Please describe this situation in which you felt authentic [inauthentic]—what happened, how you felt, etc.”*

Following the manipulation, we immediately asked the participants to estimate the degree of authenticity they felt on a 7-point scale (1 = not at all, 7 = very much) and to write down their feelings in 50 words or more, with greater being better. Finally, voice behaviors were measured in the same way as in Study 2a.

### 6.2. Results

The manipulation check procedure was the same as in Study 2a. The participants in the high-authenticity group (*M* = 6.53, *SD* = 0.62) reported feeling a higher level of authenticity than those in the low-authenticity group (*M* = 4.37, *SD* = 1.95, *t* = 8.22, *p* < 0.001, *d* = 1.49). Two independent judges also analyzed the essays on a 7-point scale (1 = low, 7 = high; *r* = 0.82). Again, the participants in the authentic group (*M* = 6.28, *SD* = 0.93) were more authentic than those in the inauthentic group (*M* = 2.13, *SD* = 1.02, *t* = 23.48, *p* < 0.001, *d* = 4.25).

Consistent with our hypothesis, the participants in the high-authenticity group (*M* = 4.15, *SD* = 0.52) reported more voice behaviors than those in the low-authenticity group (*M* = 3.54, *SD* = 0.93, *t* = 4.41, *p* < 0.001, *d* = 0.81), supporting our proposed causal chain, i.e., authenticity → voice behavior.

## 7. General Discussion

This paper used multiple methods to test multiple populations, and the results illustrated that authenticity mediated the relationship between organizational trust and voice behavior. That is, organizational trust can promote employees’ experience of authenticity at work and further lead to more voice behaviors. As we elaborate below, this paper makes the following contributions.

First, drawing on the perspective of positive psychology, we revealed the mechanism between organizational trust and voice behavior, i.e., authenticity is the key factor that mediates the relationship. This enables us to better understand why organizational trust could increase employees’ voice behaviors. Second, we used multiple measures of trust and voice as well as different populations, which guarantees the robustness and generalization of our results. Moreover, by combining with the experimental methods, we established a causal chain of this mechanism, providing a more effective examination of the psychological processes [43] underlying the relationship between organizational trust and voice behavior. Third, although Eastern cultures are characterized by interdependence and collectivism rather than the glorification of independence and self-actualization, which is quite different from Western cultures [46], this study demonstrated that Chinese people could also benefit from authenticity, especially for employees [19]. This further highlights the importance of promoting authenticity among employees. Finally, this study also extends our understanding of the antecedents of employee authenticity, i.e., enhancing organizational trust is an effective way to foster employee authenticity, which can further lead to more voice behaviors.

Despite the contribution outlined above, there are some limitations to this study. First, all participants were Chinese. More research is needed to compare the cross-cultural differences and similarities in this field. Second, drawing on the perspective of positive psychology, we only examined the mediating role of employee authenticity. Future research could include more variables, such as leader–member exchange (LMX) or employees’ self-esteem [47], to reveal more possible mechanisms underlying the relationship between organizational trust and employee voice. Third, we did not investigate the boundary conditions of the examined mediation in this study. It is possible that this mediation may be affected by employees’ individual characteristics, such as power distance orientation [48] or “dark” personality traits [48], which may limit the effects of organizational trust on employees’ voice behavior through their authenticity. Future research could include these possible moderators to make a further investigation. Fourth, following previous studies [49,50], we only investigated the mediation by using variables with aggregated scores. Future research could concentrate on each subdimension of these variables to conduct a deeper examination of our results. Finally, because this study used the hypothetical scenario technique and did not observe an actual situation in which voice behavior is needed, future research could pay more attention to other objective measures of voice behavior and generalize our findings.

## 8. Conclusions

By both using questionnaires in different samples and with different measures and experimentally manipulating variables, this study causally reveals when Chinese employees speak up in the organizational settings. Specifically, when employees experience trust from their organization, they can be their true selves and act according to their core values and beliefs (i.e., high levels of employee authenticity), thus leading to more voice behaviors. These findings highlight the importance of cultivating a trusty climate in the organization and enhancing employee authenticity in encouraging employees to speak up, which further contributes to improving organizational functioning.

## Figures and Tables

**Table 1:** Descriptive statistics and correlations among variables (*n* = 196).

	*M*	*SD*	1	2	3	4	5	6	7	8	9	10	11	12
1. Gender	_	_												
2. Age	_	_	−0.04											
3. Length of service	_	_	0.06	0.45 ^**^										
4. Educational level	_	_	−0.03	−0.22 ^**^	−0.16 ^*^									
5. Enterprise type	_	_	0.07	−0.03	0.00	−0.04								
6. Job title	_	_	−0.20 ^**^	0.27 ^**^	0.17 ^*^	0.15 ^*^	0.19 ^**^							
7. Monthly income	_	_	−0.12	0.14 ^*^	0.18 ^*^	0.28 ^**^	0.12	0.42 ^**^						
8. Marital status	_	_	−0.08	0.35 ^**^	0.36 ^**^	−0.02	0.07	0.40 ^**^	0.34 ^**^					
9. Weekly working hours	_	_	−0.04	−0.01	0.01	0.02	0.15 ^*^	0.01	−0.00	−0.04				
10. Organizational trust	3.95	0.51	−0.03	0.00	0.14	0.08	0.01	0.22 ^**^	0.17 ^*^	0.07	0.02	0.89		
11. Employee authenticity	4.75	0.86	0.14	0.05	0.17 ^*^	0.04	−0.05	0.07	0.10	0.12	−0.04	0.44 ^**^	0.92	
12. Voice behavior	3.68	0.48	−0.20 ^**^	0.17 ^*^	0.17 ^*^	0.08	−0.13	0.35 ^**^	0.23 ^**^	0.15 ^*^	0.08	0.52 ^**^	0.34 ^**^	0.74

Note. ^*^ *p* < 0.05, ^**^ *p* < 0.01. The alpha reliabilities are depicted on the diagonal.

**Table 2:** Regression results of voice behavior in Study 1a.

		Voice Behavior	
Predictors	Model 1	Model 2	Model 3
Gender	−0.11 (0.07)	−0.12 ^*^ (0.06)	−0.14 ^*^ (0.06)
Age	0.02 (0.06)	0.06 (0.05)	0.06 (0.05)
Length of service	0.08(0.05)	0.02 (0.05)	0.01 (0.05)
Educational level	0.01 (0.07)	0.004 (0.06)	0.001 (0.06)
Enterprise type	−0.08 ^**^ (0.03)	−0.07 ^**^ (0.02)	−0.07 ^**^ (0.02)
Job title	0.16 ^***^ (0.04)	0.11 ^**^ (0.04)	0.12 ^***^ (0.04)
Monthly income	0.06 (0.05)	0.03 (0.05)	0.03 (0.05)
Marital status	−0.06 (0.10)	−0.03 (0.08)	−0.04 (0.08)
Weekly working hours	0.10 (0.07)	0.09 (0.06)	0.09 (0.06)
Organizational trust		0.43 ^***^ (0.06)	0.37 ^***^ (0.06)
Employee authenticity			0.09 ^*^ (0.04)
*F*	5.38 ^***^	12.20 ^***^	11.99 ^***^
*R^2^*	0.21	0.40	0.42
Δ*R^2^*		0.19	0.02

Note. ^***^ *p* < 0.001, ^**^ *p* < 0.01, ^*^ *p* < 0.05. Unstandardized regression coefficients are presented with standard errors in parentheses.

**Table 3:** Descriptive statistics and correlations among variables (*n* = 182).

	*M*	*SD*	1	2	3	4	5	6	7	8	9	10	11
1. Gender	_	_											
2. Age	33.99	4.61	0.06										
3. Tenure	12.02	5.32	0.08	0.96 ^**^									
4. Tenure in the current company	11.43	4.78	0.15 ^*^	0.87 ^**^	0.94 ^**^								
5. Tenure in the current position	7.07	4.87	0.20 ^**^	0.39 ^**^	0.40 ^**^	0.45 ^**^							
6. Education	_	_	0.00	−0.25 ^**^	−0.26 ^**^	−0.23 ^**^	−0.08						
7. Monthly income	_	_	−0.09	0.04	0.07	0.08	0.02	0.02					
8. Marital status	_	_	−0.12	0.25 ^**^	0.27 ^**^	0.27 ^**^	0.16 ^*^	−0.18 ^*^	0.04				
9. Organizational trust	5.23	1.07	0.17 ^*^	0.06	0.07	0.07	0.03	0.03	0.10	0.07	0.97		
10. Employee authenticity	4.49	0.77	−0.03	0.15 ^*^	0.19 ^**^	0.16 ^*^	0.10	0.01	0.17 ^*^	0.05	0.16 ^*^	0.86	
11. Voice behavior	3.90	0.61	−0.03	0.12	0.16 ^*^	0.16 ^*^	0.08	0.05	0.01	0.15 ^*^	0.32 ^**^	0.42 ^**^	0.94

Note. ^*^ *p* < 0.05, ^**^ *p* < 0.01. The alpha reliabilities are depicted on the diagonal.

**Table 4:** Regressions results of voice behavior in Study 1b.

		Voice Behavior	
Predictor	Model 1	Model 2	Model 3
Gender	−0.07 (0.10)	−0.15 (0.10)	−0.12 (0.09)
Age	−0.01 (0.02)	−0.02 (0.02)	−0.02 (0.02)
Tenure in the current company	0.03 (0.02)	0.30 (0.02)	0.03 (0.02)
Tenure in the current position	0.00 (0.01)	0.002 (0.01)	−0.001 (0.01)
Education	0.10 (0.08)	0.09 (0.07)	0.07 (0.07)
Monthly income	−0.02 (0.08)	−0.05 (0.07)	−0.10 (0.07)
Marital status	0.22 (0.15)	0.16 (0.14)	0.17 (0.13)
Organizational trust		0.18 *** (0.04)	0.15 *** (0.04)
Employee authenticity			0.30 *** (0.05)
*F*	1.36	3.82 **	7.41 ***
*R^2^*	0.05	0.15	0.28
Δ*R^2^*		0.10	0.13

Note: ^***^ *p* < 0.001, ^**^ *p* < 0.01. Unstandardized regression coefficients are presented with standard errors in parentheses.

## Data Availability

The data supporting our findings can be made available from the corresponding author upon reasonable request.
